# Evaluation of left ventricular outflow tract gradient during treadmill exercise and in recovery period in orthostatic position, in patients with hypertrophic cardiomyopathy

**DOI:** 10.1186/1476-7120-6-19

**Published:** 2008-05-15

**Authors:** Rita Miranda, Carlos Cotrim, Nuno Cardim, Sofia Almeida, Luís Lopes, Maria José Loureiro, Otília Simões, Pedro Cordeiro, Paula Fazendas, Isabel João, Manuel Carrageta

**Affiliations:** 1 Hospital Garcia de Orta, Cardiology Department, Almada, Portugal; 2 Hospital da Luz, Echocardiography Department, Lisboa, Portugal

## Abstract

**Background-:**

Left ventricular outflow tract obstruction is an independent predictor of adverse outcome in hypertrophic cardiomyopathy (HCM). The classical quantification of intraventricular obstruction is performed in resting conditions in supine position, but this assessment does not reflect what happens in HCM patients (pts) in their daily activities, neither during effort nor during orthostatic recovery.

**Aim-:**

To assess intraventricular gradients with echocardiography during treadmill exercise and in the recovery period in upright position, in HCM pts.

**Methods-:**

We studied 17 HCM pts (9 males, mean age 53 ± 16 years, 11 with obstructive HCM). Each pt had 2 echocardiographic evaluations at rest (left lateral decubitus (LLD) and orthostatic position). The pts then underwent a treadmill exercise test and intraventricular gradients were measured at peak exercise and during recovery in orthostatic position.

**Results-:**

3 pts with non-obstructive HCM at rest developed intraventricular gradients during exercise. 1 pt developed this gradient only during orthostatic recovery. The mean intraventricular gradient in LLD was 49 ± 24 mmHg; in orthostatic position was 62 ± 29 mmHg (p < 0.001 versus in LLD); at peak exercise was 83 ± 35 mmHg (p < 0.001 versus supine rest); during recovery it was 96 ± 35 mmHg (p < 0.001 versus peak exercise)

**Conclusion-:**

In HCM pts the intraventricular gradient increases in orthostatic position, increases significantly during treadmill exercise and continues increasing in the recovery period in orthostatic position. This type of evaluation can help us to better understand the physiopathology, the symptoms and the efficacy of different therapeutic modalities in this disease and should be routinely used in the assessment of HCM pts.

## Introduction

Hypertrophic cardiomyopathy (HCM) is an inherited heart muscle disorder, generally characterised by asymmetric hypertrophy of left ventricular walls in the absence of another cardiac or systemic disease capable of producing a similar hypertrophy. It is a complex entity caused by a wide range of genetic, morphological and functional alterations [1-5]. A prevalence of 0.2% has been reported in the general population [6].

Approximately 25% of patients with HCM have a dynamic systolic pressure gradient in the left ventricular outflow tract caused by contact between the mitral valve leaflet(s) and the interventricular septum under resting conditions [7-11].

Outflow tract gradient in excess of 30 mmHg is an important cause of symptoms. Some authors believe that the gradient is simply a consequence of high velocity flow through the aortic valve, and hence does not represent a real obstruction to cardiac output. However, if the gradient is greater than 50 mmHg, the percentage of systolic volume ejected before the beginning of SAM is greatly reduced and this is probably responsible for patients' symptoms [12]; when severe, outflow tract gradient can cause dyspnoea, chest pain, syncope, and predisposes to the development of atrial arrhythmias [7]; it is also an independent predictor of disease progression and adverse outcome, including sudden death [13-16].

Evaluation of pts with HCM usually consists of serial echocardiographic studies in resting conditions and thus the results obtained may not reflect the heart's behavior during a patient's daily activities or the pathophysiology of this condition. The intraventricular gradient in HCM is dynamic, being greater in situations of increased LV contractility and reduced preload and afterload [17,18].

A recent study by Shah *et al *[19] demonstrates that up to two-thirds of symptomatic patients with non-obstructive HCM develop obstruction during upright exercise on a bicycle, confirming the findings in a study by Maron *et al *[20]. In this study by Shah *et al *[19], patients with exercise induced obstruction tend to have higher peak oxygen consumption than non-obstructive patients, and the gradient changes during exercise correlate with a history of syncope/presyncope. These findings have implications for the evaluation of patients with HCM, raising questions about the relation between LVOT obstruction and cardiac function, suggesting that all patients with symptomatic non-obstructive HCM at rest should have exercise stress [19].

In our department, exercise echocardiography with image acquisition during treadmill exercise (considered to reflect exercise during daily activities) is commonly used in the evaluation of pts with HCM, enabling assessment of outflow gradient during physiologic exercise and in recovery period in left lateral decubitus (LLD) [21]. In patients with obstructive HCM under resting conditions, it has been demonstrated that obstruction may increase after change of position from supine to upright [22]. In this study by Cotrim *et al*, LVOT gradient increased in orthostatic position, continued to augment at peak exercise, but after exercise the gradient decreased rapidly when measured in LLD, concluding that assessment of intraventricular gradient in recovery period in supine position does not reflect changes during effort [22]. LVOT gradient measured in recovery period in supine position does not reflect patients daily activities (pts simply don't assume supine position after an effort in their daily life) nor the pathophysiology of this condition [17,18]. Recently we found  in one patient that, after exercise, the intraventricular gradient continues to increase if we mantained the patient in orthostatic position.

The purpose of this study was to evaluate intraventricular gradients by echocardiography during isotonic treadmill exercise and immediate recovery period in upright position, in patients with hypertrophic cardiomyopathy. This study uses a different protocol from that described in a previous study [22]: intraventricular gradients are measured during immediate recovery period in orthostatic position instead of using supine position. This method tries to mimic patients daily activities.

## Methods

### Patients

We studied seventeen patients diagnosed with hypertrophic cardiomyopathy based on the echocardiographic finding of a nondilated hypertrophic left ventricle in the absence of diseases known to cause ventricular hypertrophy [3]; eleven pts with obstructive HCM due to LVOT gradient > 30 mmHg under resting conditions and six pts with non-obstructive HCM; nine males, mean age 53 ± 16 years.

The study was approved by the Ethics Committe at Garcia de Orta Hospital. All patients gave written informed consent.

### Echocardiography

Using standard techniques to obtain M-mode, two-dimensional, and Doppler measurements, each patient had two echocardiographic evaluations performed at rest: one in left lateral decubitus, with assessment of left ventricle (LV) dimensions (in systole and diastole), maximal LV wall thickness and intraventricular gradient (using continuous wave Doppler to evaluate gradients derived from Doppler velocity profiles typical of subaortic obstruction); the other in orthostatic position, with assessment of intraventricular gradients after one minute in this position.

**Figure 1:**
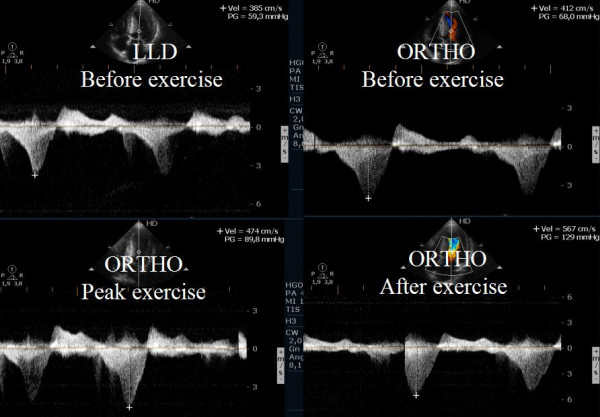
Intraventricular gradient present in all the phases of the study in one patient.

The pts then underwent a treadmill exercise test using the modified Bruce protocol, during which intraventricular gradients were measured. Data were collected at peak exercise and in recovery period in orthostatic position (Figure 1) and segmental contractility was assessed at rest and at peak exercise.

The exams were entirely recorded on videotape and partially on optical disc.

### Statistical analysis

Data are expressed as means ± standard deviation. Student's *t*-test was used for comparisons between continuous variables (systolic blood pressure, heart rate, gradients measured by continuous wave Doppler in the LV outflow tract at the various stages of the exercise stress echocardiography). A *P*-value < 0.05 was considered significant.

## Results

Baseline clinical and echocardiographic characteristics of the 17 pts with HCM are summarized in Table 1.

**Table 1:** Baseline characteristics of 17 pts with HCM

**Clinical parameters**	
Patients, n	17
Males/Females, n (%)	9 (53%)/8(47%)
Age (years-mean ± SD)	53 ± 16
NYHA II, n (%)	14 (82%)
NYHA III, n (%)	3 (18%)
Therapy, n (%)	
Beta-blocker	11 (65%)
Calcium antagonist	4 (23%)
Beta-blocker and calcium antagonist	2 (12%)
	
**Echocardiographic parameters (LLD)**	
LVOT gradient > 30 mmHg at rest, n (%)	11 (65%)
Mitral regurgitation at rest, n (%)	9 (53%)
Left ventricular end diastolic diameter (mm)	47 ± 4
Left ventricular end systolic diameter (mm)	29 ± 4
Interventricular septum thickness (mm)	17 ± 4,1
Posterior wall thickness (mm)	9 ± 2,4
Fractional shortening (%)	37 ± 5

NYHA = New York Heart Association

LVOT = left ventricular outflow tract

Left ventricular dimensions were: end-diastolic diameter 47 ± 4 mm, end-systolic diameter 29 ± 4 mm, interventricular septum thickness 17 ± 4,1 mm, posterior wall thicknes 9 ± 2,4 mm, fractional shortening 37 ± 5%.

Seven patients had trivial or mild mitral regurgitation and two moderate mitral regurgitation during rest in LLD.

On auscultation, nine patients had systolic murmurs on the left sternal border.

Fourteen patients were in NYHA class II heart failure, and three were in NYHA class III.

Eleven pts were on treatment with beta-blockers, four pts with calcium channel blockers and two pts were on treatment with calcium channel blockers and beta-blockers.

They were all in sinus rhythm and none had a history of syncope or arrhythmias (atrial fibrillation, ventricular or supraventricular tachycardia) documented in their medical records.

Table 2 shows the exercise data for the 17 pts with HCM.

**Table 2:** Exercise data in 17 pts with HCM

	**At rest**		**During exercise**	
**Exercise parameter**	**LLD**	**Orthostatic position**	**Peak exercise**	**Post exercise orthostatic**

Peak LVOT gradient, mmHg *	**49 ± 24**	**62 ± 29**	**83 ± 35**	**96 ± 35**
		(p < 0.001 versus in LLD)	(p < 0.001 versus supine rest)	(p < 0.001 versus peak exercise)
Heart rate, bpm *	**66 ± 8,6**	**74 ± 7,1**	**123 ± 11**	
Systolic blood pressure, mmHg *		**128 ± 20**	**149 ± 18**	

* Values expressed as mean ± standard deviation

LVOT = left ventricular outflow tract

Good quality Doppler flows were obtained in all cases. The values given for flow velocities are the mean of three consecutive flows.

**Figure 2:**
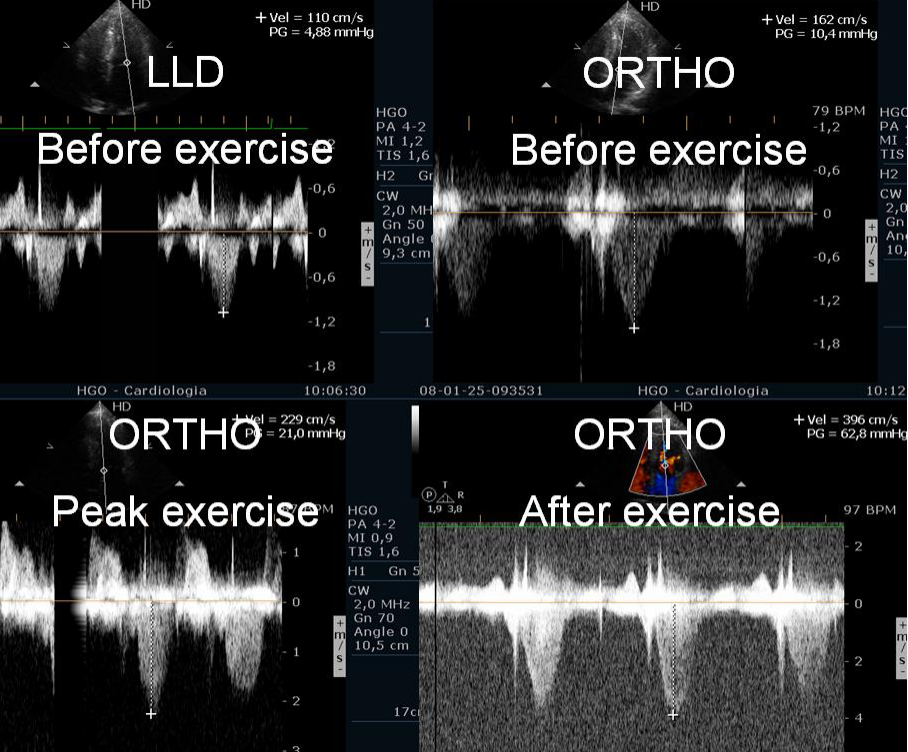
Intraventricular gradient present only in the rcovery in orthostatic position.

Three pts without resting obstruction developed intraventricular gradient during exercice (Additional File 1, 2 and 3)); one patient *developed gradient only in the recovery period in orthostatic position *(Figure 2); two pts had neither resting nor exercise induced obstruction.

The flows recorded on optical disc were measured again by the same operator and by another of the authors, and showed good intra- and inter-observer agreement with no statistically significant differences between measurements.

No segmental contractility abnormalities were detected either at rest or at peak exercise.

Pts with murmurs on initial evaluation (nine pts) increased their intensity in orthostatic position; two pts without murmur on initial assessment developed it during effort; at the end of the exercise test, all but two patients had clearly audible systolic murmurs on the left sternal border.

There were no complications. Two patients had isolated supraventricular extrasystoles and two patients isolated ventricular extrasystoles.

The exercise test was interrupted by fatigue in all patients. The mean time of exercise test was 398 ± 142 seconds.

### Study limitations

The main limitations of the study are: the small size of the sample; all patients were being medicated with beta-blockers or calcium channel blockers, which probably influenced the results; and it was not possible to obtain good quality recordings of changes in the severity of mitral regurgitation during the exercise test.

## Discussion and Conclusion

Outflow gradients under resting conditions have been shown to be independent determinants of progressive heart failure symptoms and cardiovascular mortality in HCM pts [13-16].

Heart failure symptoms and magnitude of LVOT gradients may importantly affect clinical decisions in pts with HCM [14,15,23]. Severely symptomatic drug-refractory patients with outflow obstruction, either at rest or only with physiological provocation, are potential candidates for invasive septal reduction interventions to normalize LV pressures and to improve symptoms [1].

Recent studies [19,20] depart from the traditional notion that LV outflow obstruction occurs in a minority of HCM patients, concluding that most HCM patients are predisposed to LV outflow tract obstruction because of SAM-septal contact, either at rest or with physiological exercise testing.

Exercise is the only provocative manoeuvre that is truly physiologically based and similar to conditions under which HCM patients develop symptoms [20]. The authors selected upright treadmill exercise as the most similar to pts daily activities, being a feasible methodology to define intraventricular gradients during exercise in HCM patients [22].

In the present study intraventricular gradient increases in orthostatic position, increases significantly during treadmill exercise, and continues increasing in the recovery period in upright position. These results diverge from previous studies, where intraventricular gradients decreased when measured in supine position after exercise [22]. Gradients in pts with obstructive HCM may increase after change of position from supine to upright. Orthostatism reduces preload by reducing venous return, thus increasing the LV outflow tract gradient. Dynamic isotonic exercise such as walking on a treadmill can lead to increased intraventricular gradients by increasing ventricular contractility and cardiac output [22]. Measuring intraventricular gradients using echocardiography in supine position, a routine practice in evaluation of patients with HCM does not reflect pts daily activities or the pathophysiology of this condition: supine position increases preload by increasing venous return, thus decreasing LVOT gradient and underestimating obstruction. By keeping pts in upright position after exercise, preload decreases due to sudden interruption of venous blood pumping by muscles, decreasing venous return and causing intraventricular gradients to increase. This could explain symptoms of syncope/presyncope just after exercise in pts with HMC.

The finding that intraventricular gradients increase significantly when measured during exercise has been reported by other authors using echocardiographic assessment during bicycle ergometry [19,24]. However, the present study is one of the few [22,25] with assessment carried out during treadmill exercise testing. Using treadmill exercise stress echocardiography to assess intraventricular gradients, allowed us to find that gradients increase significantly not only with exercise but also with orthostatic position before and after exercise. Intraventricular gradients assessed immediately after exercise in upright position are higher than those measured during exercise in pts with HCM.

These results suggest that measurements taken in decubitus should be viewed with some caution in assessing the severity of disease, as well as their relevance to the development of symptoms. Previous studies which evaluate LVOT gradients in pts with non-obstructive HCM during immediate recovery period in decubitus [20] may have underestimated their gradients, making it possible that the number of pts considered to have obstructive HMC may be superior to that stated.

In view of the fact that intraventricular gradients were induced in up to two-thirds of symptomatic patients with non-obstructive HCM during exercise in a recent study by Shah *et al*, and were also induced in 4 of 6 pts with non-obstructive HCM in the present study, the authors believe that the methodology described would also be useful in these patients [26], enabling to detect and quantify any obstruction developing during exercise and in recovery period in upright position, in order to determine its relevance to the patient's symptoms and prognosis.

In this study, patients were under medication with beta-blockers or calcium channel blockers, which were not suspended for safety reasons [27]. The methodology used could also be used to assess the efficacy of different types of therapy for this pathology.

Conventional exercise stress testing as used in this study, or with analysis of expired gases, is a valuable and safe aid [27,28] in evaluating and managing patients with this condition.

As a result of this small study, treatment was modified by the consulting physicians in 8 patients: associating beta-blockers in two patients medicated with verapamil, and increasing beta-blocker dosage in six patients.

The evaluation of the intraventricular gradient with exercise and in recovery period in upright position, can help us to better understand the pathophysiology, symptoms and to optimize treatment of pts with HCM.

## Authors' contributions

RM review literature and wrote the manuscript. CC performed exercise echocardiography, made clinical assessment of the patients, participate in drafting, and revised the manuscrip for important intellectual content. NC revised the manuscript for important intellectual content. SA, LL, MJL, OS, PC, PF, IJ are responsible for clinical assessment of the patients, participate in drafting, and revised the manuscrip for important intellectual content.

MC gave final approval for the manuscript. All authors read and approved the final manuscript.

## Supplementary Material

Additional file 1 Paraesternal view in one patient with non-obstructive hypertrophic cardiomyopathy. Two-dimensional paraesternal view, before exercise, showing normal morphology and funtion of left ventricle and mitral valve. Click here for file

Additional file 2 Apical 4 chamber view in one patient with non-obstructive hypertrophic cardiomyopathy. Apical 4 chamber view, before exercise showing normal flow evaluated with Doppler, showing clearly, the absence of obstruction. Click here for file

Additional file 3 Apical 4 chamber view in one patient with non-obstructive hypertrophic cardiomyopathy near peak exercise. Apical 4 chamber view, near peak exercise, showing the appearence of obstruction with Doppler. Click here for file
